# Effect of Extrusion on the Mechanical and Rheological Properties of a Reinforced Poly(Lactic Acid): Reprocessing and Recycling of Biobased Materials

**DOI:** 10.3390/ma8105360

**Published:** 2015-10-19

**Authors:** Víctor Peinado, Pere Castell, Lidia García, Ángel Fernández

**Affiliations:** 1 Fundación Aitiip, Polígono Industrial Empresarium C/Romero Nº 12, Zaragoza 50720, Spain; pere.castell@aitiip.com; 2 Tecnopackaging S.L., Polígono Industrial Empresarium C/Romero Nº 12, Zaragoza 50720, Spain; lgarcia@tecnopackaging.com; 3 Escuela de Ingeniería y Arquitectura, University of Zaragoza, Av. Maria de Luna, 3, Zaragoza 50018, Spain; afernan@unizar.es

**Keywords:** polylactic acid, nanocomposites, nanoadditive, melt strength enhancer, reprocess, rheology, mechanical, viscosity, extrusion compounding

## Abstract

The aim of this research paper is to study the behaviour of a common used biopolymer (Poly(Lactic Acid) (PLA)) after several reprocesses and how two different types of additives (a melt strength enhancer and a nanoadditive) affect its mechanical and rheological properties. Systematic extraction of extrudate samples from a twin-screw compounder was done in order to study the effect in the properties of the reprocessed material. Detailed rheological tests on a capillary rheometer as well as mechanical studies on a universal tensile machine after preparation of injected specimens were carried out. Results evidenced that PLA and reinforced PLA materials can be reprocessed and recycled without a remarkable loss in their mechanical properties. Several processing restrictions and specific phenomena were identified and are explained in the present manuscript.

## 1. Introduction

Nowadays, bio-based materials start to become a serious contestant on special applications where the price is not a limitation or where the environment impact is crucial, due to an increasing environmental consciousness [1]. One example of these families of materials is Poly(Lactic Acid) (PLA), a material derived from natural renewable sources, that can be completely biodegradable and biocompostable [2]. PLA has reasonably good optical and barrier properties compared to existing petroleum-based polymers. For instance, the permeability coefficients of CO_2_, O_2_, N_2_, and H_2_O for PLA are lower than for polystyrene (PS) but higher than poly (ethylene terephthalate) (PET). The barrier properties of PLA against organic permeants (common volatile aromas) such as ethyl acetate and d-limonene, are comparable to PET [3,4,5,6]. On the other hand, mechanically, unoriented PLA is quite brittle but possesses good strength and stiffness. Oriented PLA provides better performance than oriented PS but is comparable to PET. Tensile and flexural moduli of PLA are higher than high-density polyethylene (HDPE), polypropylene (PP) and PS, but the Izod impact strength and elongation at break values are smaller than those for these polymers [6]. Nevertheless, these last properties should be improved for their latter use on real products, and it is usual to prepare a compound together with additives (nano or micro sized) in order to improve mechanical or rheological properties [7]. Several studies have been done on this line recently, usually mixing it with cellulose nano fibers (CNF) [8,9], Sepiolite (studying its degradability [10] and mechanical properties [11,12]) or nanoclays on starch [13]. This additivation process sometimes implies the use of an extruder/compounder one or several times (depending on the equipment and the number of additives) plus the final production process that adds one extra extrusion step.

In addition to that, the cost of these biopolymers are significantly higher than commodities as PP or HDPE, which are the main the materials which PLA can replace on packaging applications. This is why to reprocess the scraps could be interesting in order to save costs and to facilitate the entrance of PLA based materials on the market.

Scrap reprocessing by extrusion-compounding is a recycling method used since years ago at the laboratory and industrial level on very different polymer materials. Evidence and studies can be found not only on non-biodegradable materials as PP [14], PP with impact modifiers [15], PP with talc [16], PP with wood fibres [17], PP with montmorillonite nanoclays [18], PE [19], PC [20], ABS [21] and PA6 [22,23], but also other biodegradable categories such as oxobiodegradable PE [24], biomaterials e.g., PBS [25] as well as on the material studied on this research, Poly(lactic acid) [26,27,28,29]. To the best of our knowledge, in these studies dedicated to reprocessing, recycling and/or degradation of PLA and its characterisation, there is no mention or specific dedicated efforts to the study of the reprocessing of PLA including the influence of different additives (different in size and properties) on it, which is the content of this paper.

In the present work, natural PLA and two other different formulations containing a melt strength enhancer and silicate nanoclays are studied systematically after its extrusion on a twin-screw compounder for 20 times, extracting one sample of 4 Kg each four extrusions in order to study the composite processing behaviour during the progression on time and number of extrusions. Thereafter/Subsequently, rheological studies are carried out with the pellets extracted as well as injected probes tested mechanically, obtaining experimental data to perform a detailed comparison among the three formulations or composite materials.

The aim of this research is to reach conclusions on the benefits and/or drawbacks of using these different additives on the reprocessed PLA, allowing industry and technicians the possibility to decide on the use of material scraps in the future with the confidence of existing evidences.

## 2. Results and Discussion

The results described on this work can be grouped by the results coming from two different tests, rheological and mechanical measurements of the samples of the different formulations.

### 2.1. Mechanical Tests

On this section, the results on Mechanical properties are shown on Table 1 and Table 2, with a final subsection for discussion of these results.

#### 2.1.1. Flexural Tests

**Figure 1:**
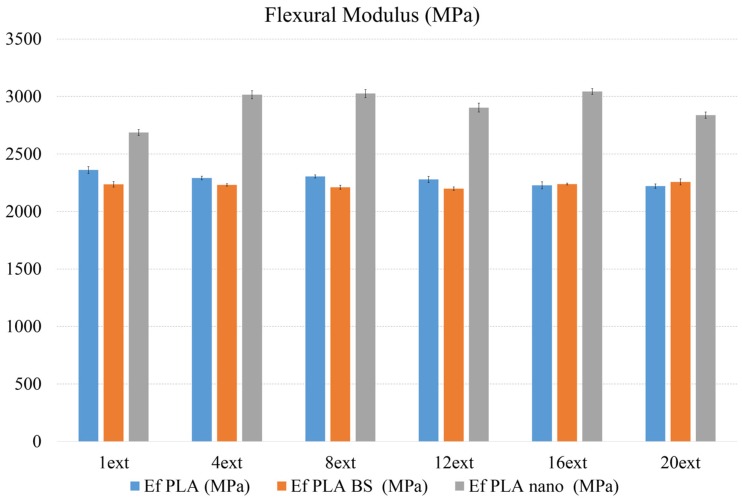
Flexural Modulus of the three formulations by each extrusion.

The results for Flexural Tests are shown as Table 1 and Figure 1.

**Table 1:** Flexural Modulus of the three formulations by each extrusion.

Natural PLA						
Series	E_f PLA_ (MPa)					
	1 ext	4 ext	8 ext	12 ext	16 ext	20 ext
$\bar{x}$	2361.02	2291.45	2304.31	2278.82	2227.60	2221.15
σ	60.00	30.58	27.17	52.74	61.96	36.94
*n [%]*	2.54	1.33	1.17	2.31	2.78	1.66
**PLA BS**						
Series	E_f PLA BS_ (MPa)					
	1 ext	4 ext	8 ext	12 ext	16 ext	20 ext
$\bar{x}$	2236.35	2231.79	2210.81	2199.02	2238.58	2257.28
σ	48.40	19.93	33.69	31.51	15.71	52.88
*n [%]*	2.16	0.89	1.52	1.43	0.70	2.34
**PLA Nano**						
Series	E_f PLA nano_ (MPa)					
	1 ext	4 ext	8 ext	12 ext	16 ext	20 ext
$\bar{x}$	2687.24	3015.48	3025.65	2903.66	3043.93	2837.88
σ	50.00	69.52	71.36	77.62	52.26	54.76
*n [%]*	1.86	2.30	2.35	2.67	1.71	1.92

#### 2.1.2. Tensile Tests

**Figure 2:**
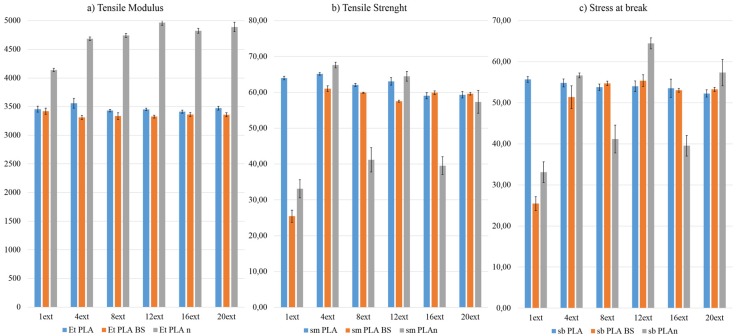
Graphical Representation of the (**a**) Tensile Modulus (E_t_); (**b**) Tensile Strength (σ_t_) and (**c**) Stress at break (σ_b_) of the three formulations by each extrusion.

The results for Tensile Tests are shown as Table 2 and Figure 2.

**Table 2:** Tensile Test results: Tensile Modulus (E_t_) Tensile Strength (σ_t_) and Stress at break (σ_b_).

Natural PLA									
	1 ext			4 ext			8 ext		
	E_t_ (MPa)	σ_t_ (MPa)	σ_b_ (MPa)	E_t_ (MPa)	σ_t_ (MPa)	σ_b_ (MPa)	E_t_ (MPa)	σ_t_ (MPa)	σ_b_ (MPa)
$\bar{x}$	3455.16	63.99	55.70	3557.98	65.17	54.86	3430.08	62.06	53.80
σ	105.06	1.02	1.39	169.26	0.76	1.91	40.98	0.84	1.53
*n [%]*	3.04	1.60	2.49	4.76	1.16	3.49	1.19	1.35	2.85
	12 ext			16 ext			20 ext		
	E_t_ (MPa)	σ_t_ (MPa)	σ_b_ (MPa)	E_t_ (MPa)	σ_t_ (MPa)	σ_b_ (MPa)	E_t_ (MPa)	σ_t_ (MPa)	σ_b_ (MPa)
$\bar{x}$	3452.41	63.09	54.03	3411.64	59.07	53.55	3471.85	59.33	52.28
σ	42.92	2.10	2.56	55.22	1.74	4.43	66.44	1.77	1.81
*n [%]*	1.24	3.33	4.73	1.62	2.94	8.27	1.91	2.99	3.46
**PLA BS**									
	1 ext			4 ext			8 ext		
	E_t_ (MPa)	σ_t_ (MPa)	σ_b_ (MPa)	E_t_ (MPa)	σ_t_ (MPa)	σ_b_ (MPa)	E_t_ (MPa)	σ_t_ (MPa)	σ_b_ (MPa)
$\bar{x}$	3421.27	25.44	25.44	3309.94	61.02	51.40	3334.46	59.89	54.73
σ	108.31	3.42	3.42	74.18	1.59	5.54	119.11	0.33	1.05
*n [%]*	3.17	13.45	13.45	2.24	2.60	10.78	3.57	0.56	1.92
	12 ext			16 ext			20 ext		
	E_t_ (MPa)	σ_t_ (MPa)	σ_b_ (MPa)	E_t_ (MPa)	σ_t_ (MPa)	σ_b_ (MPa)	E_t_ (MPa)	σ_t_ (MPa)	σ_b_ (MPa)
$\bar{x}$	3326.17	57.55	55.40	3362.31	59.90	53.04	3359.07	59.61	53.26
σ	50.97	0.51	2.87	0.07	1.04	0.90	70.55	0.78	1.03
*n [%]*	1.53	0.89	5.18	0.71	1.74	1.69	2.10	1.30	1.93
**PLA Nano**									
	1 ext			4 ext			8 ext		
	E_t_ (MPa)	σ_t_ (MPa)	σ_b_ (MPa)	E_t_ (MPa)	σ_t_ (MPa)	σ_b_ (MPa)	E_t_ (MPa)	σ_t_ (MPa)	σ_b_ (MPa)
$\bar{x}$	4137.82	33.10	33.10	4684.56	67.64	56.67	4742.92	41.16	1.53
σ	58.28	5.09	5.09	64.84	1.58	1.20	67.55	6.81	0.42
*n [%]*	1.41	15.37	15.37	1.38	2.33	2.12	1.42	16.54	27.78
	12 ext			16 ext			20 ext		
	E_t_ (MPa)	σ_t_ (MPa)	σ_b_ (MPa)	E_t_ (MPa)	σ_t_ (MPa)	σ_b_ (MPa)	E_t_ (MPa)	σ_t_ (MPa)	σ_b_ (MPa)
$\bar{x}$	4966.33	64.50	64.50	4822.84	39.54	39.54	4890.39	57.36	2.48
σ	91.60	2.69	2.69	84.66	4.99	4.99	167.33	6.39	0.41
*n [%]*	1.84	4.17	4.17	1.76	12.63	12.63	3.42	11.14	16.68

#### 2.1.3 Discussion of Mechanical Properties Results

Regarding the Flexural Modulus and Tensile Modulus, both properties share similar trends (see Figure 1 and Figure 2 respectively). The number of extrusions does not affect flexural nor tensile moduli on the natural PLA or the PLA BS, this last one being a melt-strengthening additive which does not provide any benefit on the mechanical modulus on the final product, as expected.

It is has been shown that Flexural and Tensile Moduli on nanoreinforced PLA show an increasing trend on their values while the number of extrusions increases, effects that can be justified due to a better dispersion of the nanoclays on the polymer matrix after several reprocesses.

When studying Tensile Strength and Stress at break, we can see that the three materials show a very brittle behaviour, PLA nano samples being the most brittle. All probes broke on their maximum value of Tensile strength (same value for stress at break), followed by PLA BS, that allows a little deformation after the maximum tensile stress and finally continuing with the natural PLA, which is the more ductile and easily deformed material.

Elongation at break was not reported because the dispersion of the results was too high. These values can be seen in Table 3.

**Table 3:** Dispersion of elongation at break values. Elongation at break (E_tb_), v [%] Coefficient of variation in %.

E_tb_ (v [%])	Ref	4 ext	8 ext	12 ext	16 ext	20 ext	Mean
PLA	12.41	24.63	23.98	18.39	15.10	11.91	17.73767
PLA BS	24.31	5.22	9.52	6.63	3.72	7.59	9.498661
PLA nano	24.51	12.10	27.78	9.40	23.18	16.68	18.94274

### 2.2. Rheological Tests

The studies were carried out at three varying temperatures that comprise the processing temperature range or processing window of PLA: 170, 180, 190 °C; the three different formulations (Natural PLA, PLA BS and PLA Nano), for each of the six samples extracted. The results are listed in an Annex on detail and the curves summarizing the data are shown below and the values reported are averaged values of three measured curves.

#### 2.2.1. Natural PLA

**Figure 3:**
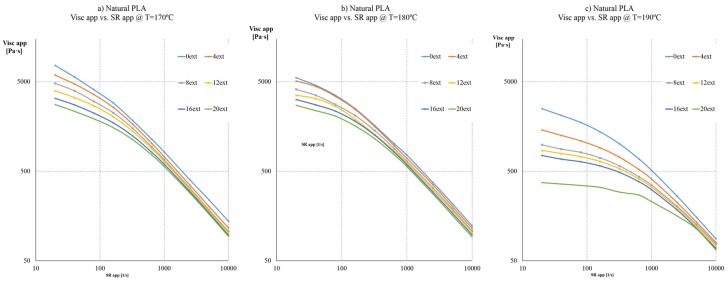
Apparent viscosity curves at different apparent Shear rates of each extrusion sample for: (**a**) Natural PLA at 170 °C; (**b**) Natural PLA at 180 °C; (**c**) Natural PLA at 190 °C.

Results on natural PLA show expectable behaviour of the biopolymer as shown on Figure 3. As the PLA is extruded, its viscosity decreases due to polymer chain degradation. A noticeable decrease of viscosity at higher temperatures (190 °C) should be mentioned; around this temperature, the degradation due to reprocessing is more remarkable: for instance, at 320 s^−1^, the viscosity decreases a 70%, from 1009.62 to 292.90 Pa·s.

#### 2.2.2. PLA with Melt Strength Enhancer

**Figure 4:**
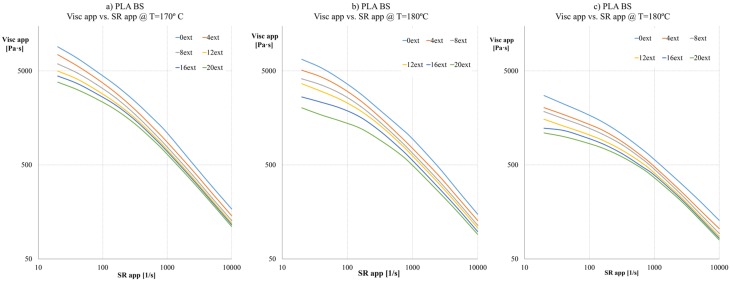
Apparent viscosity curves at different apparent Shear rates of each extrusion sample of: (**a**) PLA BS at 170 °C; (**b**) PLA BS at 180 °C; (**c**) PLA BS at 190 °C.

Viscosity curves (Figure 4) on the case of PLA blended with Biostrength (PLA BS) show a similar behaviour compared to Natural PLA. The decrease in viscosity values at 190 °C is less aggressive (in this case at 320 s^−1^, the viscosity decreases 42%, from 1057.38 to 608.12 Pa·s). The nominal values are higher than natural PLA as expected. These results affirm the applicability of this kind of additives for their use on regrinded or reprocessed PLA. As a general conclusion of the PLA BS performance, it can be stated that if the number of extrusions is increased (>8 extrusions), the melt strength enhancer (Biostrength) manages to better retain the viscosity values of the material. Thus, the reprocessing step of the material containing BS is significantly enhanced.

#### 2.2.3. PLA with Silicate Nanoclays

**Figure 5:**
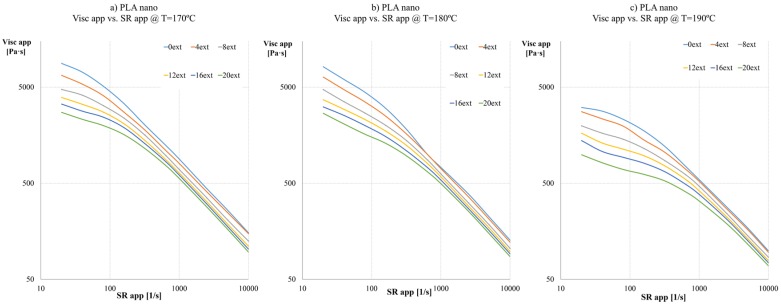
Apparent viscosity curves at different apparent Shear rates of each extrusion sample of: (**a**) PLA nano at 170 °C; (**b**) PLA nano at 180 °C; (**c**) PLA nano at 190 °C.

For the third formulation PLA reinforced with nanoadditives, we can appreciate a behaviour midway between the previous two. Again, looking at Figure 5, the viscosity curves with nanoadditives (PLA nano) show a comparable trend to previous cases. The decrease in the values at 190 °C is noticeable, but not as noteworthy as on natural PLA; in this third case, at 320 s^−1^, the viscosity decreases a 56%, from 1205.06 to 527.34 Pa·s. The nominal values are closer to the ones of PLA BS than natural PLA, but lower overall. It has been noticed that for less than eight extrusions, and in a high range of temperatures T ≥ 180 °C, the use of nanoadditives instead of Biostrength leads to higher viscosity values, due to the reinforcement effect as a result of a better dispersion of nanoadditives.

#### 2.2.4. Comparisons between the Three Formulations as a Function of Temperature

We should note that, in this section, we are going to study the batches behaviour comparing the percentage of variation (decrease) in apparent viscosity.

##### At a Temperature of 170 °C

**Figure 6:**
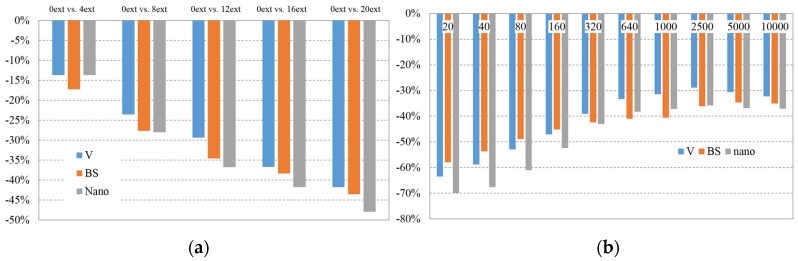
(**a**) Average Variation of Viscosity value at 170 °C by number of extrusions (compared *versus* 0 extrusions batch); (**b**) Viscosity value variation at 170 °C by shear rate (compared 0 extrusions *versus* 20 extrusions batch).

Figure 6a shows the average variation of viscosity values by extrusion batch. This decrease is contrasted *versus* the values of the original material (0 extrusions). At a temperature of 170 °C, the decreasing behaviour of natural PLA (V) appears more lineal, but in the case of PLA BS (BS), the decreasing is faster at the beginning (<12 extrusions) and it slows down at the end. The behaviour of PLA nano (Nano) is similar than natural PLA until four extrusions but accelerates from extrusion number 8.

Figure 6b shows the variation of viscosity between 0 and 20 extrusions batches at different shear rates. All batches show great decrease on viscosity after 20 extrusions. It can be interpreted as the decrease of viscosity due to degradation. The differences between the original values of viscosity and the ones on the latter extrusions are higher on lower shear rates. It means that the shear thinning effect is reduced as the number of extrusions increases due to a narrower molecular weight distribution of the recycled polymer. Therefore, the impact of recycling on processes with high shear rates as injection moulding is lower than on processes where the low shear stress is dominant.

##### At a Temperature of 180 °C

At a temperature of 180 °C, again the decreasing behaviour of natural PLA is more lineal, but now this decrease is smaller than at lower temperatures. In the case of PLA BS, it decreases linearly with the number of extrusions, being even greater than the decreasing of PLA nano. It should be mentioned that there is a reduced decrease in viscosity of natural PLA at a low number of extrusions for the usual processing temperature of PLA.

**Figure 7:**
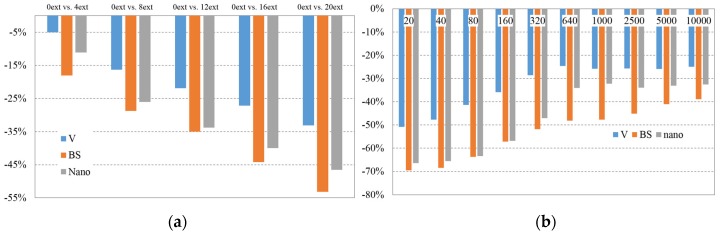
(**a**) Average Variation of Viscosity value @ 180 °C by extrusion batch (compared *versus* 0 extrusions batch); (**b**) Viscosity value variation by shear rate [compared 0 extrusions *versus* 20 extrusions batch] @ 180 °C.

On Figure 7b, natural PLA shows a better behaviour than at 170 °C for low shear rates. PLA BS and PLA Nano show a mayor viscosity decrease at lower shear rates than natural PLA. At higher shear rates (>640 s^−1^), the three materials show near constant viscosity decrease. It is lower for natural PLA (<25%), medium for PLA Nano (<35%) and higher for PLA BS (between 40% and 50%). The stabilisation of viscosity decrease at higher shear stress increases the confidence to use these material formulations for injection moulding processing.

##### At a Temperature of 190 °C

At a temperature of 190 °C, the linear decreasing behaviour of natural PLA remains, though in this case the influence of the high temperature is clearer and the decrease in viscosity is much higher than at lower temperatures. For the cases of PLA BS and PLA Nano, the behaviour keeps similar than at 180 °C. It should be mentioned that in the current case, the reduced decrease of viscosity on a low number of extrusions belongs to PLA Nano, proving the presence of nanoadditives, which compensates matrix degradation. The opposite effect appears on a high number of extrusions, where the relative viscosity decrease of PLA Nano shows an increasing tendency.

**Figure 8:**
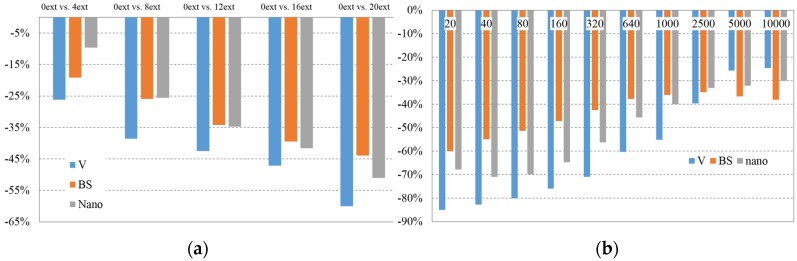
(**a**) Average Variation of Viscosity value @ 190 °C by extrusion batch (compared *versus* 0 extrusions batch); (**b**) Viscosity value variation by shear rate [compared 0 extrusions *versus* 20 extrusions batch] @ 190 °C.

On Figure 8b, the same behaviour on PLA Nano can be noted/observed, proving that the presence of nanoparticles stabilises the viscosity decrease in relation with the shear rate with no dependence of the temperature. Natural PLA decrease of properties on almost all shear rates is accentuated when high temperatures affect the material.

## 3. Experimental Section

### 3.1. Materials

The materials used for this research are Natural PLA Ingeo^®^ Natureworks 2003D, with a melt flow rate (MFR) of 6 g/10 min (2.16 kg, 210 °C) and specific density of 1.24 g/cm^3^ (Blair, NE, USA); it is a transparent general purpose extrusion grade that is used naturally or as part of a formulated blend. This is a high molecular weight biopolymer grade that processes easily on conventional extrusion equipment.

The melt strength enhancer was Arkema Biostrength^®^ (La Garenne-Colombes, France) 700 [30,31,32,33]. This copolymer is selected as an additive due to its recommendation to be used with PLA for applications where improvements in melt strength and ease of processability are desired, and can be used to compensate for losses in melt strength when using high levels of regrind PLA. Its physical form is white powder, with a particle size of a 2% max on 40 Mesh, a specific gravity of 1.17 g/cm^3^ and a bulk density of 0.45 g/cm^3^. It was included in a concentration of 4 wt %.

Finally, the nanoadditive selected was an experimental silicate nanoclay produced from Avanzare Innovación Tecnológica (Logroño, La Rioja, Spain), DIB 17, with a length of 2 microns and an average thickness of 10 nm, on a 3 wt % concentration. Previous internal developments of our research group, demonstrated that mechanical properties of PLA were improved when this nanoadditive was integrated on the matrix.

### 3.2. Equipment

For the preparation of the formulations, a 26 mm twin-screw Coperion ZSK 26 compounder machine (Coperion, Baden-Wurtemberg, Stuttgart, Germany) with two Brabender gravimetric feeders (Brabender, Duisburg, North Rhine-Westphalia, Germany) was used. For the injection moulding of the probes, a JSW 85 EL II electric injection machine (Japan Steel Works LTD, Tokyo, Japan) with a 32 mm diameter reciprocating screw (Japan Steel Works LTD, Tokyo, Japan) was used, together with a mould following standards ISO 178 and ISO 527 for the shapes of the tensional and flexural probes.

Regarding testing machines: for rheological measurements, a capillary rheometer CEAST SmartRHEO 20 from Instron^®^ (Cerdanyola, Barcelona, Spain) was used and for mechanical tests a Zwick/Roell universal tensile machine with 10 KN maximum capacity (Zwick, Ulm, Baden-Württemberg, Germany).

### 3.3. Methods

The first part of the work started with the preparation of the three formulations on the compounder. For this purpose, PLA was dried in advance inside a dehumidifier for more than 12 h at a temperature of 55 °C, below its glass transition temperature (*T_g_*), and ensured that the moisture content was below 0.025% (250 ppm). Then, 30 Kg of each three formulations were extruded 20 times, and one sample of 4 Kg was extracted for each of the 4 extrusions (6 different samples in total). The conditions of extrusions were a temperature profile 175 > 180 > 185 > … > 185 > 175 °C and a screw speed of 180 rpm with a low shear rate screw profile.

With the batches already prepared, the flexural and tensile probes (10 for each test and batch) were injected at a screw speed of 131 rpm, with a temperature profile increasing from 170 °C at the hopper, to 185 °C at the barrel, 190 °C at the nozzle nearby and up to 200 °C at the end tip of the nozzle. Dosage and filling pressure were varied for each formulation injected. A packing pressure of 100 MPa was applied.

With all the required preparation processes finished, the second part of the work was carried out concerning the rheological and mechanical properties measurement. On the one hand, viscosity measurements were performed at 170, 180 and 190 °C for all the 18 batches collected, and measurements on shear rates between 20 and 10,000 s^−1^ on 40, 80, 160, 320, 640, 1000, 2500, 5000 s^−1^ were done. Furthermore, the diameter of the die, 1 mm and its length, 20 mm, being the detection of each point of the curve automatic where the plateau was reached inside a tolerance of 1%.

On the other hand, mechanical measurements were done first with the flexural probes. Flexural tests were conducted under ambient conditions using a crosshead speed of 2 mm/min. 10 specimens of each batch were essayed in a three-point bending configuration with a distance between supports of 57 mm. Flexural modulus was calculated between deformations 0.025% and 1.2%. Then, the Tensile tests were carried out with a starting speed of 500 mm/min, a pre-load of 0.1 MPa and calculation speeds for Tensile modulus of 1 mm/min, between 0.05% and 0.45% of strain, and Yield strength of 3 mm/min.

## 4. Conclusions

The present work has demonstrated the reprocessability and the recyclability of novel biobased reinforced materials and the effect of different additives on its performance. The authors have studied the effect of reprocessing conditions on the mechanical and rheological properties of neat and reinforced PLA materials. Silicate nanoclays have been selected as reinforcing additives due to their good behaviour at high shear rates, ease of processing and dispersability, which also combine the possibility of regrinding and/or reprocessing. The incorporation of a melt strength has also been evaluated as it is generally used in the formulation of biobased compositions to enhance its processability.

A systemic methodology for sample extraction during extrusion of the different compositions (up to 20 reprocessing extrusions) has been used for the complete characterisation of all the formulations studied. The obtained results demonstrated that despite the fact that both PLA and reinforced PLA materials showed a decrease in the viscosity during each reprocessing step, no remarkable loss in their mechanical properties is observed. Tensile value modulus for neat PLA of 3455 MPa are maintained in 3471 MPa after 20 extrusions indicating that no negative effects can be attributed to reprocessing. Nanoreinforced materials showed an unexpected behaviour as the tensile modulus increased from 4137 up to 4890 MPa after 20 extrusions. This effect is attributed to the improvement on the dispersion of the nanofiller after each process.
